# Implementation of a machine learning application in preoperative risk assessment for hip repair surgery

**DOI:** 10.1186/s12871-022-01648-y

**Published:** 2022-04-23

**Authors:** Yu-Yu Li, Jhi-Joung Wang, Sheng-Han Huang, Chi-Lin Kuo, Jen-Yin Chen, Chung-Feng Liu, Chin-Chen Chu

**Affiliations:** 1 Department of Anesthesiology, Chi Mei Medical Center, Tainan, Taiwan; 2 Department of Medical Research, Chi Mei Medical Center, Tainan, Taiwan

**Keywords:** Hip surgery, Risk assessment, Machine learning

## Abstract

**Background:**

This study aims to develop a machine learning-based application in a real-world medical domain to assist anesthesiologists in assessing the risk of complications in patients after a hip surgery.

**Methods:**

Data from adult patients who underwent hip repair surgery at Chi-Mei Medical Center and its 2 branch hospitals from January 1, 2013, to March 31, 2020, were analyzed. Patients with incomplete data were excluded. A total of 22 features were included in the algorithms, including demographics, comorbidities, and major preoperative laboratory data from the database. The primary outcome was a composite of adverse events (in-hospital mortality, acute myocardial infarction, stroke, respiratory, hepatic and renal failure, and sepsis). Secondary outcomes were intensive care unit (ICU) admission and prolonged length of stay (PLOS). The data obtained were imported into 7 machine learning algorithms to predict the risk of adverse outcomes. Seventy percent of the data were randomly selected for training, leaving 30% for testing. The performances of the models were evaluated by the area under the receiver operating characteristic curve (AUROC). The optimal algorithm with the highest AUROC was used to build a web-based application, then integrated into the hospital information system (HIS) for clinical use.

**Results:**

Data from 4,448 patients were analyzed; 102 (2.3%), 160 (3.6%), and 401 (9.0%) patients had primary composite adverse outcomes, ICU admission, and PLOS, respectively. Our optimal model had a superior performance (AUROC by DeLong test) than that of ASA-PS in predicting the primary composite outcomes (0.810 vs. 0.629, *p* < 0.01), ICU admission (0.835 vs. 0.692, *p* < 0.01), and PLOS (0.832 vs. 0.618, *p* < 0.01).

**Conclusions:**

The hospital-specific machine learning model outperformed the ASA-PS in risk assessment. This web-based application gained high satisfaction from anesthesiologists after online use.

**Supplementary Information:**

The online version contains supplementary material available at 10.1186/s12871-022-01648-y.

## Introduction

A comprehensive preoperative evaluation can enhance the quality of patient care and is associated with a reduced mortality rate [1–3]. In preoperative evaluation clinics, the anesthesiologist must evaluate the patient's medical history and laboratory data, determine the patient’s physical status, and draw up a preoperative management plan in a limited time. After the initial assessment, the anesthesiologist discusses these risks with the patient and the surgical team [2]. In the case of emergency or urgent surgeries, all these tasks must be achieved with high efficiency [4].

The incidence of hip fractures is gradually increasing because the aging population is continually growing, and it is causing a heavy burden on society [5, 6]. Almost all patients with hip fractures would require surgical treatment, and anesthesia intervention is inevitable. These patients are mostly geriatric and have many comorbidities.

Several estimation tools have been used to help physicians assess operative risks [7–9]. These models range from simple scoring systems, such as the American Society of Anesthesiologist-Physical Status (ASA-PS) [10], to more complex calculators, such as the Surgical Risk Preoperative Assessment System (SURPAS) and the American College of Surgeons National Surgical Quality Improvement Program (ACS-NSQIP®) Surgical Risk Calculator [11–13]. Although the former is easy to use, it ignores many important parameters, such as sex, age, comorbidities, and laboratory data; while the latter includes more parameters, it requires tedious work. For instance, the ACS-NSQIP, which is an open-access web-based online tool that is currently gaining worldwide acceptance, involves manually entering 19 to 21 patient-specific variables [13–15]. In a busy medical ecology that requires efficiency, developing an automated preoperative evaluation system that incorporates multiple parameters is imminent.

We aimed to develop a machine learning-based application that can assist anesthesiologists in assessing specific adverse outcomes for patients required to undergo hip repair surgery. We hypothesized that a machine learning algorithm, which includes variables such as patient demographics, comorbidities, laboratory data, and anesthesiologists’ initial assessment, may have superior performance in risk assessment than the ASA-PS scoring. Through the assistance of the data-driven application, anesthesiologists will be able to effectively evaluate patients and precisely inform them of the operative risks, allowing them to have shared decision-making in a real-world medical domain.

## Methods

### Study design

We established a multidisciplinary team including anesthesiologists, data scientists, and information engineers for this retrospective study. Data were extracted from the Chi-Mei Medical Center’s hospital information systems (HIS) database to build the AI prediction models. We deleted the medical record number and all types of personal identification of each patient to protect their privacy.

**Fig. 1:**
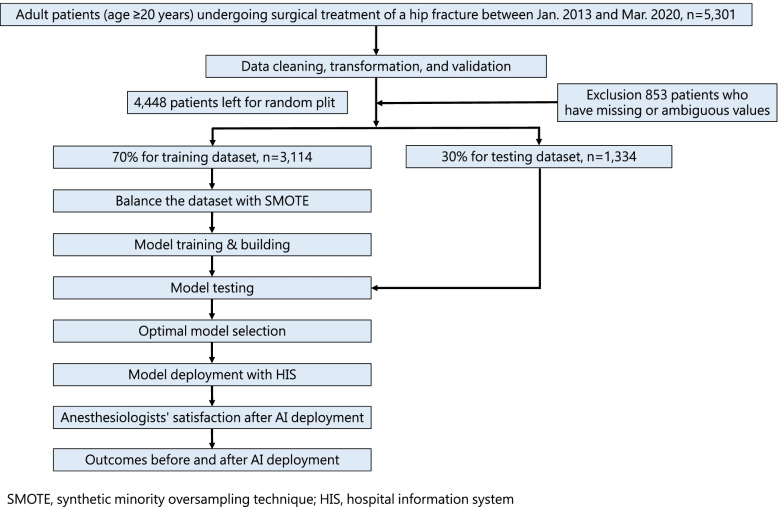
Flow chart of study

All methods were carried out per relevant guidelines and regulations of Chi Mei Medical Center. The construction of the database was approved by the institutional review board (Serial No. 10906–008). Informed consent was waived because of the retrospective design of the study, which only involved secondary analysis of existing data and had no direct patient contact. After machine learning model training and performance testing, the optimal models were deployed into the existing HIS to assist anesthesiologists in performing preoperative risk assessment for patients with hip fractures (Fig. 1).

### Study setting and patient selection

Adult patients aged 20 years and above who underwent surgical treatment of a hip fracture at Chi Mei Medical Center and its 2 branch hospitals in Tainan City, Taiwan, from January 1, 2013, to March 31, 2020, were selected based on current procedural terminology (CPT) codes. The CPT codes for enrollment included hip fixation codes (27,235, 27,236, 27,244, and 27,245), hemiarthroplasty (27,125), and total hip arthroplasty (27,130) with an admission diagnosis of hip fracture (ICD-9 codes 820.x or ICD-10 codes S70-S79). Patients with incomplete perioperative data, those whose body weight was 30 kg and below, and those whose height was 100 cm and below were excluded from the study. A total of 5,301 patients were initially reviewed for this study, but only 4,448 patients were included after considering the exclusion criteria.

### Feature variables

Using a similar method from previous studies [16–18], established clinical importance and clinical expert opinion were used to select 22 preoperative variables from the HIS dataset as inputs to the algorithm for calculating the risk of adverse events of interest. The feature variables retrieved from the HIS database include (1) patient demographics (e.g., age, sex, body mass index, and smoking status); (2) preoperative comorbidities (e.g., heart diseases such as coronary artery disease, congestive heart failure, old myocardial infarction, previous cerebral stroke, dialysis use, presence of COPD; (3) laboratory values (e.g., serum sodium, white blood cell count, hematocrit, platelet count, creatinine, blood urea nitrogen, creatinine, albumin, and prothrombin time); and (4) operative and anesthetic variables (e.g., ASA-PS status, mode of anesthesia, and the anticipated arterial line and central venous pressure monitoring). These features were integrated into the algorithm for machine learning. To develop the models, the patients were randomly split into a training cohort (70%) and a testing cohort (30%). This separation helped ensure that the test set was kept completely independent from the training set.

### Study outcomes

This study’s primary outcome was a composite of postoperative adverse events, including (1) in-hospital mortality and death within 48 h after discharge (discharge death code); (2) acute stroke (ICD-9-CM codes 430 to 436 and 997.02, and ICD-10-CM codes I609, I619, I6789); (3) acute myocardial infarction (AMI) (ICD-9-CM code 410, and ICD-10-CM codes I21 or I23); (4) acute respiratory failure (ICD-9-CM codes 518.81 to 518.82, 518.84, and 518.5, and ICD-10-CM codes J96); (5) sepsis (ICD-9-CM codes 038, and ICD-10-CM codes R65); (6) acute liver failure (ICD-9-CM codes 570, and ICD-10-CM codes K7200); and (7) acute renal failure (ICD-9-CM codes 584.9 and ICD-10-CM codes 570, and ICD-10-CM codes with ICD-10-CM codes K7200); and (7) dialysis code (ICD-9-CM codes 584). Moreover, postoperative intensive care unit (ICU) admission and prolonged length of stay (PLOS) were set as secondary outcomes.

### Machine learning algorithms

The models were trained with 7 machine learning algorithms consisting of (1) logistic regression, (2) random forest, (3) k nearest neighbor (KNN), (4) support vector machines (SVM), (5) light gradient boosting machine (light GBM), (6) eXtreme gradient boosting (XGBoost), and (7) deep learning of multilayer perception (MLP). To address the issue of class imbalance in the training cohort, the synthetic minority oversampling technique was utilized. Python programming language with scikit.learn machine learning library was used for model building. Grid searching with 5-fold cross-validation for hyperparameter tuning for each algorithm was performed to obtain the optimal models. The goal of the algorithms is to predict the primary and secondary outcomes.

### Model performance

Each model was used to predict the test set. The specificity, sensitivity, accuracy, and area under the receiver operating characteristic curve (AUROC) were calculated and the models’ predictive performances were compared based on the AUROC value.

### Implementation of web-service application to HIS

The optimal algorithm with the highest AUROC was used to build a web-based application, then integrated into the HIS for pre-anesthetic patient evaluation.

### Anesthesiologist satisfaction score after AI-assisted risk assessment

After each completion of the AI-assisted risk assessment, the system automatically requests its users to grade their level of satisfaction from 0 (most dissatisfied) to 5 (highly satisfied). The first month, which was done online, was employed as the benchmark reference to compare the changes in the satisfaction of anesthesiologists during the study period from July 2020 to April 2021.

### Incidence of adverse outcomes before and after web-based application deployment in HIS

We deployed the AI-assisted risk assessment application online beginning July 1, 2020. To assess whether the developed application improved the medical outcomes, we compared the incidences of primary composite adverse outcomes, ICU admission, and PLOS before (July 2019 to April 2020) and after (July 2020 to April 2021) AI-assisted risk assessment.

### Statistical analysis

Descriptive statistical analysis of the data was performed using SPSS 13.0 for Windows (SPSS, Inc., Il, USA). Continuous variables were defined as the means and standard deviations or medians and ranges. Countable variables were defined with numbers and percentages. The models’ predictive performances were compared with each other and with conventional ASA-PS risk stratification based on the AUROC value using the Delong test [19]. A series of one-way analyses of variance were conducted to examine the differences in the satisfaction score among the five-month groups. After, Tukey's honestly significant difference post hoc test was performed to detect the intergroup differences. The level of significance was set at a *p*-value less than 0.01.

## Results

### Demographics

From January 1, 2013, to March 31, 2020, a total of 5,301 adult patients who had hip fractures and received hip repair surgery under GA or NA were identified. After removing excluded patients, data from 4,448 patients underwent analysis. From this, 3,114 patients (70%) were randomly allocated for training the machine learning models, and 1,334 patients (30%) were set as the validation cohort (Fig. 1).

Patient demographics and characteristics of the training and testing data sets are summarized in Table 1. The mean age of the patients was 65.3 years, and they are mostly females (57.6%). Approximately 70.8% of them were stratified as ASA-PS 3 status. The event rates were 2.3% (*N* = 120), 3.6% (*N* = 160), and 9.0% (*N* = 401) for composite primary adverse events, ICU admission, and PLOS, respectively. As shown in Table 1, patients with primary composite adverse events and ICU admission were older, mostly males, had anemia, longer prothrombin time, and activated partial thromboplastin time (aPTT), and had comorbidities such as chronic respiratory diseases, cancer, heart disease, dementia, or advanced chronic kidney disease (stage 4 and 5).

**Table 1:** Demographic data of study patients

Demographics	Total	Primary composite adverse outcomes			ICU admission			Prolonged hospital stay		
		No	Yes	*P*-Value	No	Yes	*P*-Value	No	Yes	*P*-Value
Cases,n (%)	4448 (100)	4346 (97.7)	102 (2.3)		4288 (96.4)	160 (3.6)		4047 (91.0)	401 (9.0)	
Age mean (SD)	65.3 (18.6)	65.1 (18.6)	70.9 (18.4)	0.003	65.2 (18.6)	67.7 (20.2)	0.129	65.8 (18.4)	60.0 (20.5)	< 0.001
Sex, male, n (%)	1885 (42.4)	1831 (42.1)	54 (52.9)	0.037	1801 (42.0)	84 (52.5)	0.011	1661 (41.0)	224 (55.9)	< 0.001
BMI, mean (SD)	23.8 (4.2)	23.8 (4.2)	23.3 (4.7)	0.339	23.8 (4.2)	23.2 (4.7)	0.114	23.7 (4.2)	24.2 (4.6)	0.049
Smoking, n (%)	618 (13.9)	599 (13.8)	19 (18.6)	0.21	594 (13.9)	24 (15.0)	0.768	545 (13.5)	73 (18.2)	0.011
Emergency, n (%)	2186 (49.1)	2129 (49.0)	57 (55.9)	0.202	2106 (49.1)	80 (50.0)	0.889	2062 (51.0)	124 (30.9)	< 0.001
ASA-PS classification										
ASA-PS 1, n (%)	61 (1.4)	60 (1.4)	1 (1.0)	< 0.001	60 (1.4)	1 (0.6)	< 0.001	60 (1.5)	1 (0.2)	< 0.001
ASA-PS 2, n (%)	1031 (23.2)	1026 (23.6)	5 (4.9)		1026 (23.9)	5 (3.1)		987 (24.4)	44 (11.0)	
ASA-PS 3, n (%)	3150 (70.8)	3073 (70.7)	77 (75.5)		3041 (70.9)	109 (68.1)		2858 (70.6)	292 (72.8)	
ASA-PS 4–5, n (%)	206 (4.6)	187 (4.3)	19 (18.6)		161 (3.7)	45 (28.1)		142 (3.5)	64 (16.0)	
Anesthesia										
GA, n (%)	4191(94.2)	4097 (94.3)	94 (92.2)	0.49	4039 (94.2)	152 (95.0)	0.797	3806 (94.0)	385 (96.0)	0.135
CVC, n (%)	267 (6.0)	242 (5.6)	25 (24.5)	< 0.001	227 (5.3)	40 (25.0)	< 0.001	198 (4.9)	69 (17.2)	< 0.001
Arterial line n (%)	1367 (30.7)	1301 (29.9)	66 (64.7)	< 0.001	1275 (29.7)	92 (57.5)	< 0.001	1197 (29.6)	170 (42.4)	< 0.001
Laboratory data										
ALT, mean (SD)	29.3 (59.1)	28.0 (29.1)	82.6(338.3)	0.107	27.6(27.8)	73.1(273.4)	0.037	26.4 (24.0)	58.4 (179.1)	< 0.001
eGFR, mean (SD)	76.2 (30.8)	76.3 (30.3)	71.2 (45.8)	0.267	76.2 (30.0)	75.7 (47.5)	0.898	74.7 (28.5)	90.9 (45.3)	< 0.001
Hb, mean (SD)	10.8 (1.6)	10.9 (1.6)	9.7 (1.5)	< 0.001	10.9 (1.6)	9.9 (1.5)	< 0.001	10.9 (1.6)	10.3 (1.6)	< 0.001
aPTT, mean (SD)	28.0 (4.3)	27.9 (4.2)	31.4 (8.2)	< 0.001	27.9 (4.2)	30.1 (7.2)	< 0.001	27.9 (4.2)	29.0 (5.7)	< 0.001
PT, mean (SD)	10.9 (1.6)	10.8 (1.4)	12.5 (4.1)	< 0.001	10.8 (1.4)	11.9 (3.5)	< 0.001	10.8 (1.5)	11.4 (2.4)	< 0.001
Platelet, mean(10^3^) (SD)	231.9(90.6)	232.4(89.1)	212.2(139.8)	0.151	231.4(86.6)	246.5 (164.5)	0.249	226.6(81.6)	285.3(144.3)	< 0.001
Comorbidity										
Respiratory, n (%)	457 (10.3)	425 (9.8)	32 (31.4)	< 0.001	414 (9.7)	43 (26.9)	< 0.001	376 (9.3)	81 (20.2)	< 0.001
Diabetes, n (%)	1079 (24.3)	1049 (24.1)	30 (29.4)	0.266	1038 (24.2)	41 (25.6)	0.751	1005 (24.8)	74 (18.5)	0.005
Hypertension, n(%)	1795 (40.4)	1744 (40.1)	51 (50.0)	0.057	1732 (40.4)	63 (39.4)	0.861	1683 (41.6)	112 (27.9)	< 0.001
Liver disease, n (%)	174 (3.9)	166 (3.8)	8 (7.8)	0.061	163 (3.8)	11 (6.9)	0.078	149 (3.7)	25 (6.2)	0.017
Malignancy, n (%)	408 (9.2)	387 (8.9)	21 (20.6)	< 0.001	386 (9.0)	22 (13.8)	0.057	361 (8.9)	47 (11.7)	0.078
Heart disease, n (%)	565 (12.7)	541 (12.4)	24 (23.5)	0.002	531 (12.4)	34 (21.2)	0.001	508 (12.6)	57 (14.2)	0.382
CKD-stage 1, n (%)	1477 (32.5)	1412 (32.5)	35 (34.3)	< 0.001	1386 (32.3)	61 (38.1)	< 0.001	1225 (30.3)	222 (55.4)	< 0.001
CKD-stage 2, n (%)	1783 (40.1)	1757 (40.4)	26 (25.5)		1746 (40.7)	37 (23.1)		1703 (42.1)	80 (20.0)	
CKD-stage 3, n (%)	865 (19.4)	849 (19.5)	16 (15.7)		837 (19.5)	28 (17.5)		816 (20.2)	49 (12.2)	
CKD-stage 4, n (%)	177 (4.0)	166 (3.8)	11 (10.8)		162 (3.8)	15 (9.4)		156 (3.9)	21 (5.2)	
CKD-stage 5, n (%)	176 (4.0)	162 (3.7)	14 (13.7)		157 (3.7)	19 (11.9)		147 (3.6)	29 (7.2)	
Stroke, n (%)	467 (10.5)	450 (10.4)	17 (16.7)	0.058	445 (10.4)	22 (13.8)	0.217	430 (10.6)	37 (9.2)	0.432
Dementia, n (%)	377 (8.5)	353 (8.1)	24 (23.5)	< 0.001	347 (8.1)	30 (18.8)	< 0.001	333 (8.2)	44 (11.0)	0.074
Schizophrenia, n (%)	32 (0.7)	31 (0.7)	1 (1.0)	0.525	31 (0.7)	1 (0.6)	1.000	25 (0.6)	7 (1.7)	0.021

Primary composite adverse outcomes included in-hospital mortality (and death in 48 h after discharge), sepsis, acute myocardial infarction, acute stroke, respiratory, liver and renal failure.

Abbreviations: *BMI* body mass index, *ASA-PS* American society of anesthesiologist-physical status, *GA* general anesthesia, *CVC* central venous catheter, *ALT* alanine aminotransferase, *eGFR* estimated glomerular filtration rate, *Hb* hemoglobin, *CKD* chronic kidney disease

Correlation analysis (Table 2) identified the correlation between each feature's outcome. For composite primary adverse outcomes, the most relevant features were anticipated intraoperative arterial line and central venous catheter monitoring, preoperative hemoglobin, and respiratory comorbidity; for ICU admission, the most relevant features were ASA-PS status, arterial line central venous pressure monitoring, and preoperative hemoglobin; and for PLOS, the key factors included emergency surgery, ASA-PS, central venous monitoring, ALT, eGFR, P.T., and comorbid of respiratory disease.

**Table 2:** The correlation coefficients between each feature and each outcome

Features	Outcome		
	Composite adverse	ICU admission	Prolonged hospital stay
Age	0.054	0.034	-0.080
Sex	0.033	0.040	0.086
Body Mass Index	-0.014	-0.028	0.031
Smoking	0.021	0.006	0.039
Emergency	0.021	0.003	-0.115
ASA-PS	0.094	0.158	0.146
General anesthesia	-0.014	0.006	0.024
CVC	0.119	0.154	0.148
Arterial line	0.113	0.112	0.080
ALT	0.029	0.055	0.144
eGFR	-0.025	-0.010	0.127
Hemoglobin	-0.102	-0.110	-0.103
aPTT	0.078	0.060	0.052
Prothrombin time	0.096	0.088	0.106
Platelet	-0.056	-0.016	0.120
Respiratory disease	0.106	0.106	0.103
Diabetes Mellitus	0.025	0.014	-0.026
Hypertension	0.033	0.002	-0.065
Liver disease	0.031	0.030	0.038
Malignancy	0.061	0.031	0.028
Heart disease	0.050	0.050	0.014
CKD stage	0.031	0.023	-0.097
Stroke	0.053	0.041	-0.009
Dementia	0.083	0.071	0.028

Abbreviations: *ASA-PS* American Society of Anesthesiologist-physical status, *CVC* Central venous catheter, *ALT* Alanine aminotransferase, *eGFR* Estimated glomerular filtration rate, *aPTT* activated partial thromboplastin time, *CKD* Chronic kidney disease

### Prediction of primary composite adverse outcomes

In the machine learning prediction of primary composite adverse outcomes such as in-hospital mortality, mortality within 48 h after discharge, and major organ injury, the sensitivity by logistic regression, SVM, and lightGBM all reached 0.710; the SVM had the highest specificity (0.716) followed by KNN (0.711) (Table 3). All models, except KNN, achieved high AUROCs which were between 0.734 (XGBoost, 95% C.I.: 0.636 ~ 0.831) and 0.794 (Logistic regression, 95% C.I.: 0.718 ~ 0.869) (Appendix 1).

**Table 3:** Predictive performance of machine learning algorithms on primary composite adverse outcomes^*^

Algorithm	Accuracy	Sensitivity	Specificity	AUROC (95%CI)
Logistic Regression	0.699	0.710	0.699	0.794 (0.718–0.869)
Random Forest	0.690	0.677	0.690	0.776 (0.704–0.848)
SVM	0.716	0.710	0.716	0.768 (0.677–0.860)
KNN	0.706	0.516	0.711	0.644 (0.542–0.746)
lightGBM	0.703	0.710	0.703	0.786 (0.706–0.867)
MLP	0.691	0.677	0.692	0.777 (0.684–0.859)
XGBoost	0.638	0.645	0.638	0.734 (0.636–0.831)

^*^Total 102 patients had primary composite adverse outcomes (Primary composite adverse outcomes included in-hospital mortality (and death in 48 h after discharge), sepsis, acute myocardial infarction, acute stroke, respiratory, liver and renal failure

Abbreviations: *AUROC* area under receiver operating characteristic curve, *CI* confidence interval, *SVM* support vector machine, *KNN* K nearest neighbor, *light GBM* light gradient boosting machine, *MLP* multi-layer perception, *XGBoost* extreme gradient boosting

### Prediction of postoperative ICU admission

As shown in Table 4, in the prediction of ICU admission, MLP (0.812) and logistic regression (0.792) had the highest sensitivity. Further, logistic regression (0.791), lightGBM (0.769), and the random forest (0.760) had the highest specificity. Moreover, logistic regression, lightGBM, and the random forest had the highest accuracy (between 0.760 to 0.791). Except for KNN and SVM, all models had AUROCs between 0.825 (XGBoost, 95% C.I.: 0.772 ~ 0.878) and 0.856 (Logistic regression, 95% C.I.: 0.804 ~ 0.908) (Appendix 2).

**Table 4:** Predictive performance of machine learning algorithms on ICU admission^*^

Algorithm	Accuracy	Sensitivity	Specificity	AUROC (95%CI)
Logistic Regression	0.791	0.792	0.791	0.856 (0.804–0.908)
Random Forest	0.760	0.750	0.760	0.844 (0.788–0.899)
SVM	0.706	0.708	0.706	0.730 (0.648–0.812)
KNN	0.658	0.542	0.662	0.630 (0.549–0.712)
lightGBM	0.769	0.771	0.769	0.842 (0.788–0.896)
MLP	0.734	0.812	0.731	0.829 (0.779–0.885)
XGBoost	0.709	0.708	0.709	0.825 (0.772–0.878)

^*^ICU admission: 160 patients

Abbreviations: *ICU* intensive care unit, *AUROC* area under receiver operating characteristic curve, *CI* confidence interval, *SVM* support vector machine, *KNN* K nearest neighbor, *light GBM* light gradient boosting machine, *MLP* multi-layer perception, *XGBoost* extreme gradient boosting, *CI* confidence interval

### Prediction of prolonged length-of-stay (PLOS)

The results of model prediction on PLOS are shown in Table 5. The random forest and lightGBM had higher sensitivity (0.783 and 0.767, respectively) and specificity (0.783 and 0.774, respectively) than other algorithms. Moreover, the random forest had the best performance with the highest AUROCs (0.854; 95% C.: 0.818 ~ 0.890) (Table 5 and Appendix 3).

**Table 5:** Predictive performance of machine learning algorithms on prolonged length-of-stay*

Algorithm	Accuracy	Sensitivity	Specificity	AUROC (95% CI)
Logistic Regression	0.745	0.742	0.745	0.831 (0.791–0.871)
Random Forest	0.778	0.783	0.778	0.854 (0.818–0.890)
SVM	0.651	0.650	0.651	0.730 (0.679–0.780)
KNN	0.643	0.625	0.644	0.681 (0.627–0.736)
lightGBM	0.773	0.767	0.774	0.853 (0.815–0.892)
MLP	0.727	0.741	0.726	0.824 (0.791–0.871)
XGBoost	0.747	0.750	0.747	0.837 (0.797–0.876)

^*^ Prolonged length-of-stay: hospital stay longer than that of 90 percentiles in the validated cohort. Prolonged hospital stay: 401 patients

Abbreviations: *AUROC* area under receiver operating characteristic curve, *SVM* support vector machine, *KNN* K nearest neighbor, *light GBM* light gradient boosting machine, *MLP* multi-layer perception, *XGBoost* extreme gradient boosting, *CI* confidence interval

### The performance of machine learning models and ASA-PS

**Fig. 2:**
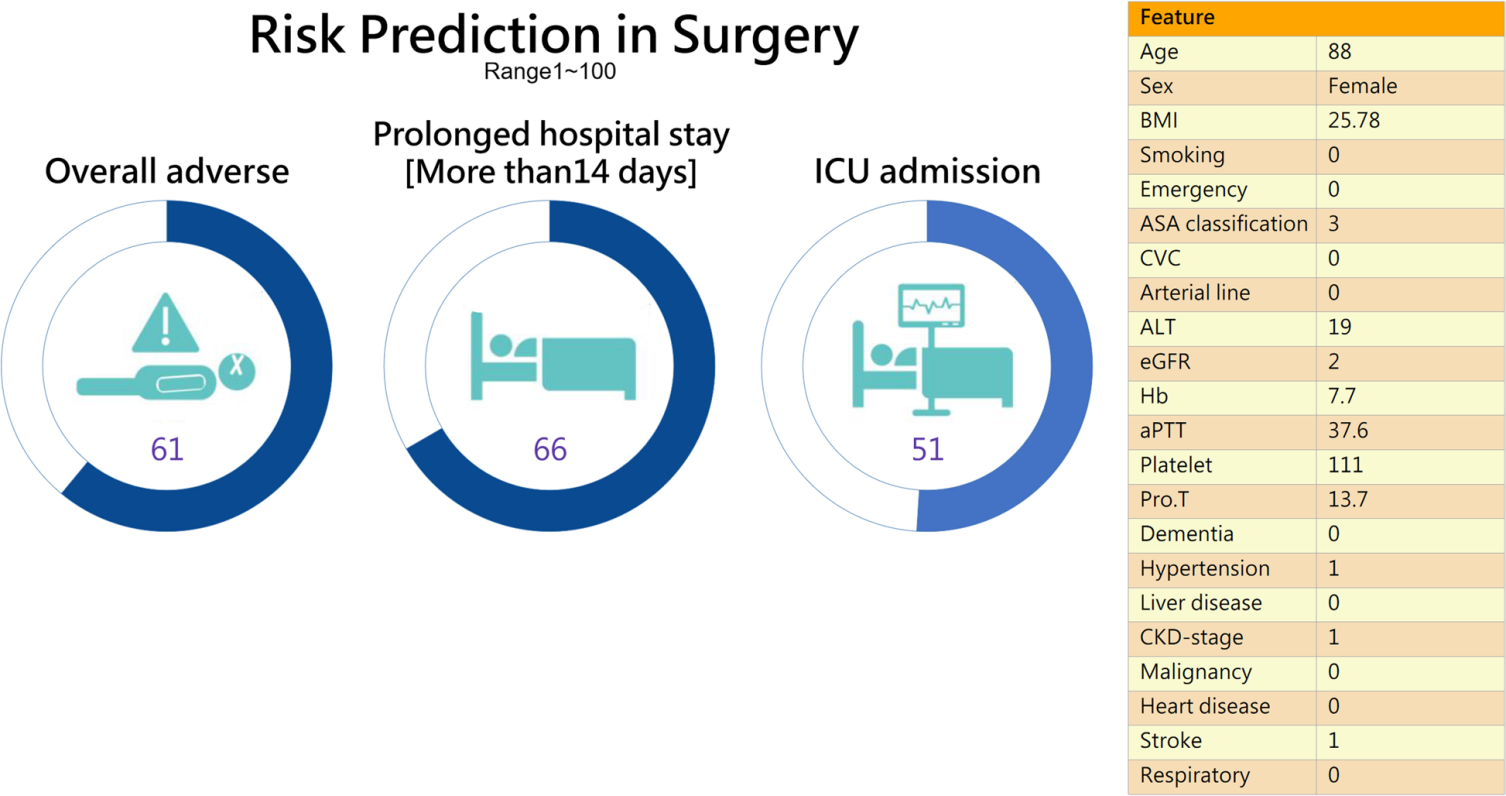
A Snapshot of the web-based application in the hospital information system

Based on the results, an AI web-based application was constructed using logistic regression for adverse outcomes and ICU admission, and the random forest for PLOS. Figure 2 shows a snapshot of the AI web service application for predicting adverse outcomes in the pre-anesthetic visit clinic.

The results in Table 6 indicate that the machine learning AI web-based application had superior AUROC scores (Delong test, *P* < 0.001) than ASA-PS stratification in terms of primary composite adverse outcomes (0.776 vs. 0.629), ICU admission (0.844 vs. 0.629), and PLOS (0.854 vs. 0.618).

**Table 6:** Comparison of AI models with ASA-PS for primary composite adverse outcomes, ICU admission and prolonged length of hospital stay

**Outcome**	**Model**	**Accuracy**	**Sensitivity**	**Specificity**	**AUC (95%CI)**	***P*-value**^%^
**Composite adverse**	ASA	0.326	0.896	0.262	0.629 (0.590–0.668)	< 0.001
	^b^AI model	0.538	0.903	0.529	0.794 (0.718–0.869)	
**ICU admission**	ASA	0.931	0.240	0.958	0.692 (0.645–0.738)	< 0.001
	^b^AI model	0.979	0.240	0.979	0.856 (0.804–0.908)	
^a^**PLOS**	ASA	0.336	0.909	0.279	0.618 (0.582–0.654)	< 0.001
	^c^AI model	0.649	0.908	0.624	0.854 (0.818–0.890)	

^a^ *PLOS* Prolong length of hospital-stay

^b^ Using logistic regression for AI models for primary composite adverse outcomes and ICU admission

^c^ Using Random Forest for AI model for PLOS

^%^ Delong test

### The incidences of adverse outcomes before and after AI-assisted application deployment

Table 7 demonstrates the demographics and incidences of adverse outcomes in 545 and 500 patients before and after implementing the online web-based application. There was no statistically significant decrease in the incidence of primary composite adverse events (3.3 vs. 1.6%, *p* = 0.117) or ICU admission (4.4 vs. 2.4%, *p* = 0.109) after the web-based application was initially employed for clinical use.

**Table 7:** The incidences of major adverse outcomes before and after AI web-based application online use

Demographics	Before AI^a^	After AI	*P*-Value^*^
	2019/07–2020/04	2020/07–2021/04	
	*N* = 545	*N* = 500	
Age, mean (SD)	65.1 (18.5)	64.7 (17.9)	0.238
Sex, female, n (%)	306 (56.1)	252 (50.4)	0.072
Sex, male, n (%)	239 (43.9)	248 (49.6)	
ASA-PS classification			
ASA-1, n (%)	9 (1.7)	6 (1.2)	0.207
ASA-2, n (%)	141 (25.9)	104 (20.8)	
ASA-3, n (%)	376 (69.0)	368 (73.6)	
ASA-4–5, n (%)	19 (3.5)	22 (4.4)	
Primary composite outcome, n (%)	18 (3.3)	8 (1.6)	0.117
ICU admission, n (%)	24 (4.4)	12 (2.4)	0.109
PLOS^c^, n (%)	50 (9.2)	54 (10.8)	0.439

^*^ two-tailed student t test or chi-squared test, as appropriate

^a^ Application online was used since 2020/07/01

^b^ *PLOS* prolonged length-of-stay

### The satisfaction score web-based application from anesthesiologists

**Fig. 3:**
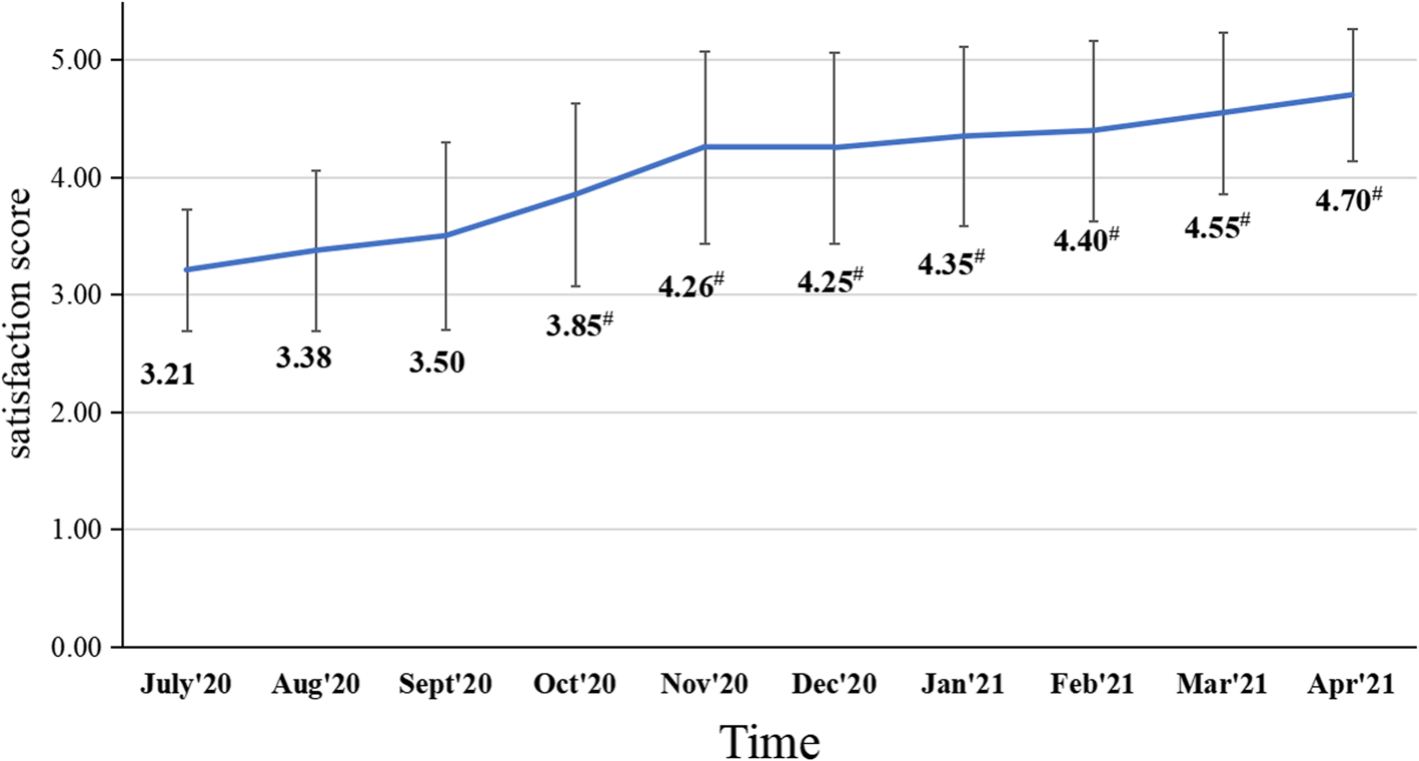
Anesthesiologists’ satisfaction ratings of the web-based application since its implementation

The AI Assist Application was launched on July 1, 2020, and by April 30, 2021, a total of 500 patients were evaluated under the assistance of AI. Figure 3 illustrates that the satisfaction score rose from 3.21 ± 0.51 (1^st^ month online) to 4.70 ± 0.56 (10^th^ month). The score was significantly higher starting in the 4^th^ month after the application was launched (*P* < 0.01).

## Discussion

In this retrospective study using the HIS database, machine learning methods were applied in our hospital-specific real-world medical domain to assist anesthesiologists in their preoperative risk assessment for patients required to undergo hip fracture repair surgery in terms of primary composite adverse outcomes (mortality and major organ injuries), the need for ICU admission and PLOS. The newly developed AI assist application was found to have significantly higher sensitivity, specificity, accuracy, and performance (AUROC) than that of the ASA-PS, the traditional and most widely used risk stratification method. The major strength of this study is its successful integration of the AI-assisted app into the hospital’s HIS system. The novel contribution of this study is that the machine learning algorithm empowered the ASA-PS scoring, allowing more specific prognostic assessments for patients undergoing hip surgery. Moreover, this online app is user-friendly and received high satisfaction scores from anesthesiologists who used it.

Machine learning can simultaneously deal with numerous variables by building statistical models based on outliers and nonlinear interactions among variable [20, 21]. To use our application, the anesthesiologist, after evaluating the patient, first inputs the ASA-PS followed by the mode of anesthesia and whether an arterial line or a ventral venous catheter is anticipated. Then, the AI assist application automatically captures 22 features, which are important independent risk factors [22, 23], from the HIS. All the anesthesiologist has to do is click the calculation button. The application will start to run and calculate the risk scores for in-hospital mortality, ICU admission, and PLOS. The results of the calculation are then displayed on the computer screen.

The results of this study demonstrated that modern AI computer systems can not only collect and display data but can also play an active role in assisting physicians with their risk assessments, allowing them to make a shared decision with the patient or their family members.

Previous machine learning applications in hip fracture have demonstrated high potential for automated detection of hip fractures on radiographs and hip fracture risk prediction [24–26]. Further, recent studies have tried to build a precise model for mortality of hip fracture surgery [27]*.* This current research not only developed an AI-based risk predictive model that performed better than ASA-PS in terms of risk assessment for hip fracture surgery but also successfully incorporated the application into the hospital’s existing HIS, allowing it to be used in daily practice. It is important for hospitals to establish a more reliable and available model of risk assessment for patients who need to undergo hip fracture surgery. A precise model could help improve physicians’ shared decision-making with their patients and assist in evaluating the need for critical care monitoring after surgery. Machine learning techniques can integrate a large amount of data already captured in the HIS, which offers a prediction model with better predictive performance and facilitates automation.

This research does not suggest the discontinuation of the ASA-PS system nor does it refute the need for human intelligence; instead, it aims to add a machine-learning algorithm to facilitate efficiency in preoperative risk assessment. Anesthesiologists need to judge the patient’s ASA physical status and decide whether an arterial line or CVC is anticipated perioperatively. The AI assist application takes the above information, in conjunction with patient data captured from the HIS, to calculate the risk of adverse events.

Machine learning algorithms have been proven to more accurately assess the risks associated with anesthesia and surgery [28, 29]. A study published by Ehlers et al. [28] used the insurance claims database and calculated the Naïve Bayes algorithm to predict the risk of postoperative complications and showed superiority to Charlson's comorbidity index. In the present study, more variables were adapted, and the data were used for the training of 7 algorithms. A recent study by Li et al. [30] reported AI prediction using the random forest algorithm to predict 1-year mortality after hip repair surgery. They collected data from 1,330 and 744 patients to train and validate the AI algorithm, respectively. In the present study, data from 3,114 patients for training and 1,334 for validation were included. Because the sample size is greater, the statistical power is therefore stronger. Moreover, other than mortality, the risk of ICU admission and PLOS were also estimated.

Aside from the widely accepted ASA-PS system, there are also some pre-anesthetic risk stratification tools being used in hospitals, such as the ACS-NSQUIP, an open-access online tool based on the logistic model [31]. Our AI application shares some similarities with ACS-NSQIP. Both tools have some variables in common, such as age, sex, ASA, emergency surgery, and CPT procedure code. The ACS-NSQIP is calculated based on logistic regression, while our AI application used seven algorithms, including logistic regression, for machine learning. After comparing the AUROC of these algorithms, the best algorithm was selected to build the AI prediction tool. Moreover, although there is now an open-excess ACS-NSQIP, it has not been built into the Chi Mei Medical Center HIS system yet. Therefore, the present study did not include this as a reference comparison. Further comparative studies are very worth conducting in the future.

Although anesthesiologists have affirmed the online application of this study's app, we still have not observed a significant effect on reducing the incidence of adverse outcomes, ICU admission, and PLOS. It may require a longer observation time and a larger population of patients to justify the efficacy of this web-based application.

### Limitations

Some limitations are inherent in retrospective machine learning projects using hospital-specific databases. First, the accuracy of prediction algorithms at specific hospitals may be limited by hospital-specific factors. However, the methodology could theoretically be generalized to similar hospitals with similar patient races or under similar health insurance systems. Second, this study was dependent on the correctness of the ICD-9- or ICD-10-CM coding while identifying study cases, comorbidities, and complications. These codes were reviewed and validated by auditors of medical records for the insurance system to ensure the accuracy of the claims; however, there is still the possibility of miscoding and misclassifying some diseases and conditions. Third, the study’s data were extracted from a single medical institution and its 2 branch hospitals; thus, an underlying referral bias might have existed. Therefore, to obtain a more generalizable result, external validation using patient cohorts from other institutions is required. Fourth, since this is a retrospective study, further validation in a prospective manner to demonstrate predictive capability is needed. Fifth, postoperative mortality in the study’s model was limitedly captured from in-hospital complications and in-hospital death or death within 48 h after discharge. Our models demonstrated relatively short-term mortality or adverse events because some patients, especially those who had complications or were dissatisfied with the surgical service, might have transferred to other hospitals without a referral. Therefore, the study endpoints were limited to the in-hospital period. Sixth, non-geriatric hip fracture may have different pathophysiologic mechanisms from geriatric hip fractures and may require different assessment tool. In our preliminary analysis, we subgrouped study patients into those above 50 (*n* = 3551) and below 50 years (*n* = 897), however, this number of non-geriatric patients was insufficient to support machine learning. Therefore, the current research cannot meet this need.

## Conclusions

The AI assist application developed using a machine learning algorithm was found to be helpful for anesthesiologists in evaluating the risks associated with hip surgery more efficiently and accurately than the traditional ASA-PS stratification method. Although this study may be limited by hospital-specific factors, it could still be generalized to hospitals with similar patient races and comparable health insurance systems. Moreover, this web-based application gained a high satisfaction score from anesthesiologists, which implies an urgent need for automated artificial intelligence assistance in preoperative risk assessment.

## Supplementary Information

**Additional file 1. Appendix 1.** ROC curves for each machine learning model after testing the validation datasets on the risk of adverse events prediction.

**Additional file 2. Appendix 2.** ROC curves for each machine learning model after testing using the validation datasets on intensive care unit admission prediction.

**Additional file 3. Appendix 3.** ROC curves for each machine learning model after testing using the validation datasets on prolonged hospital stay prediction.

## Data Availability

The data that support the findings of this study are available from the corresponding author on reasonable request. (Email:chinchen.chu@gmail.com).

## Abbreviations

SVM: Support vector machine
KNN: K nearest neighbor
light GBM: A light gradient boosting machine
MLP: Multilayer perception
XGBoost: An extreme gradient boosting
AUROC: Area under the receiver operating characteristic curve
CI: Confidence interval
BMI: Body mass index
ASA-PS: American Society of Anesthesiologist-physical status
GA: General anesthesia
CVC: Central venous catheter
ALT: Alanine aminotransferase
eGFR: Estimated glomerular filtration rate
Hb: Hemoglobin
CKD: Chronic kidney disease
