# Supplementary figures and images for: Implementation of a machine learning application in preoperative risk assessment for hip repair surgery
Source: BMC Anesthesiol. 2022 Apr 23;22:116. doi: 10.1186/s12871-022-01648-y (PMC9034633; doi:10.1186/s12871-022-01648-y)

ROC curve(Overall adverse)

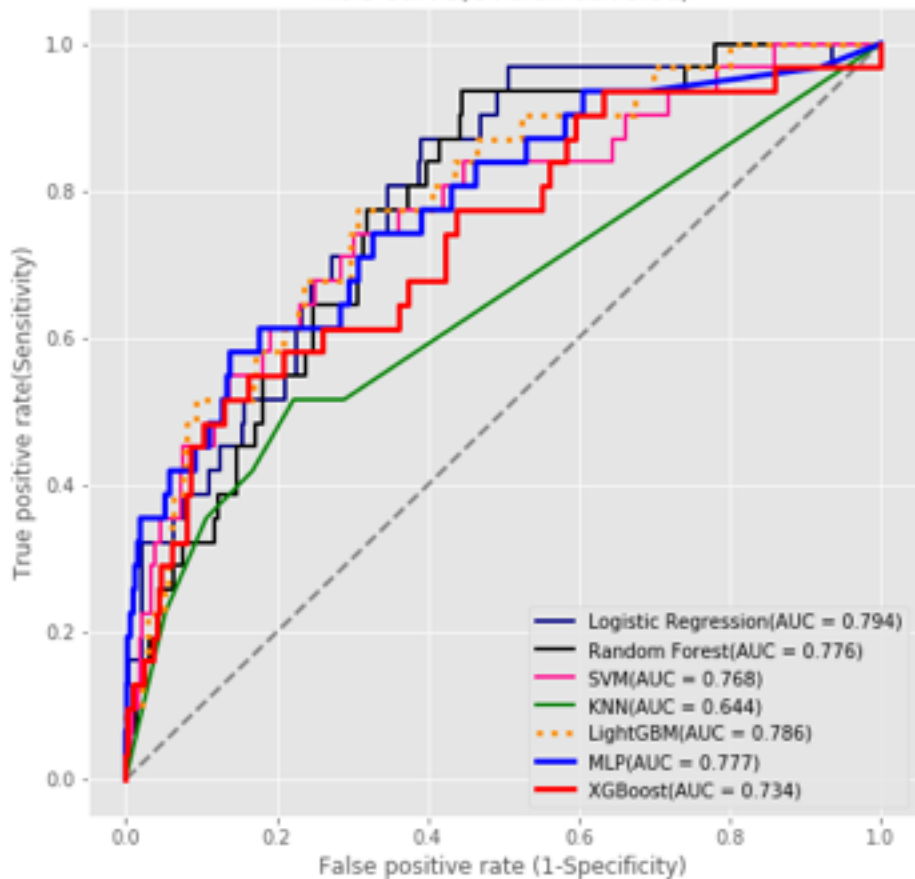

Supplement: Supplementary file 1 — Additional file 1. Appendix 1. ROC curves for each machine learning model after testing the validation datasets on the risk of adverse events prediction. [file 12871_2022_1648_MOESM1_ESM.pdf]

ROC curve(ICU admission)

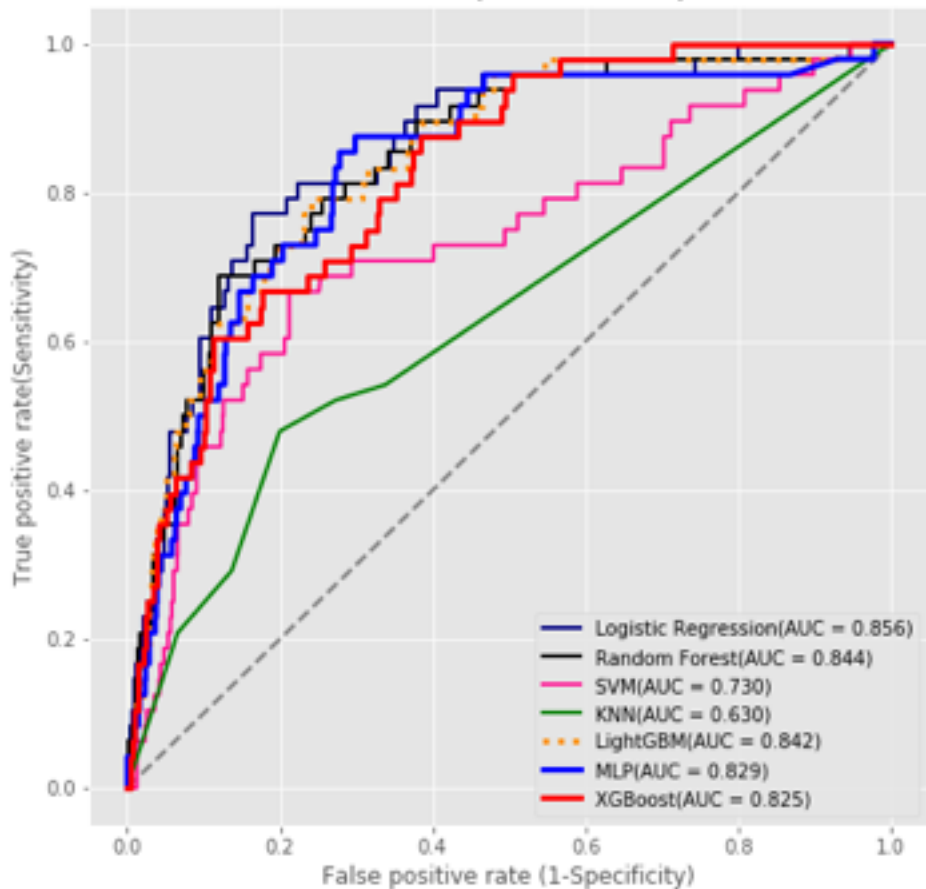

Supplement: Supplementary file 2 — Additional file 2. Appendix 2. ROC curves for each machine learning model after testing using the validation datasets on intensive care unit admission prediction. [file 12871_2022_1648_MOESM2_ESM.pdf]

ROC curve(Prolonged hospital stay)

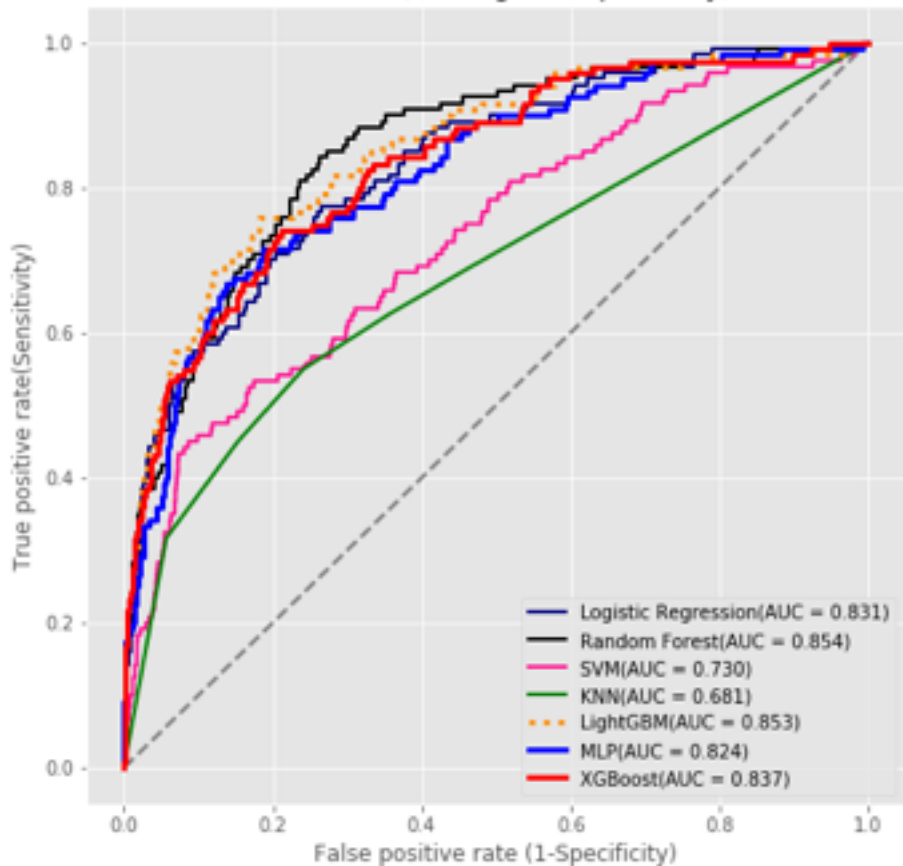

Supplement: Supplementary file 3 — Additional file 3. Appendix 3. ROC curves for each machine learning model after testing using the validation datasets on prolonged hospital stay prediction. [file 12871_2022_1648_MOESM3_ESM.pdf]
